# Ceramide present in cholangiocarcinoma-derived extracellular vesicle induces a pro-inflammatory state in monocytes

**DOI:** 10.1038/s41598-023-34676-w

**Published:** 2023-05-12

**Authors:** Barbara Oliviero, Michele Dei Cas, Aida Zulueta, Roberta Maiello, Alessandro Villa, Carla Martinelli, Elena Del Favero, Monica Falleni, Linda Montavoci, Stefania Varchetta, Dalila Mele, Matteo Donadon, Cristiana Soldani, Barbara Franceschini, Marcello Maestri, Gaetano Piccolo, Matteo Barabino, Paolo Pietro Bianchi, Jesus M. Banales, Stefania Mantovani, Mario U. Mondelli, Anna Caretti

**Affiliations:** 1 Division of Clinical Immunology - Infectious Diseases, Department of Research, Fondazione IRCCS Policlinico San Matteo, Pavia, Italy; 2 Department of Health Sciences, University of Milan, Milan, Italy; 3 Neurorehabilitation Unit of Milan Institute, Istituti Clinici Scientifici Maugeri IRCCS, Milan, Italy; 4 Department of Molecular Medicine, University of Pavia, Pavia, Italy; 5 Department of Medical Biotechnology and Translational Medicine, University of Milan, Milan, Italy; 6 Pathology Division, Health Sciences Department, University of Milan, Milan, Italy; 7 Department of Biomedical Sciences, Humanitas University, Pieve Emanuele, Milan, Italy; 8 Department of Hepatobiliary and General Surgery, IRCCS Humanitas Research Hospital, Rozzano, Milan, Italy; 9 Laboratory of Hepatobiliary Immunopathology, IRCCS Humanitas Research Hospital, Rozzano, Milan, Italy; 10 Division of General Surgery 1, Fondazione IRCCS Policlinico San Matteo, Pavia, Italy; 11 General Surgery Unit, Department of Health Sciences, San Paolo Hospital, University of Milan, Milan, Italy; 12 Department of Liver and Gastrointestinal Diseases, Biodonostia Health Research Institute-Donostia University Hospital, University of the Basque Country (UPV/EHU), San Sebastian, Spain; 13 National Institute for the Study of Liver and Gastrointestinal Diseases (CIBERehd, “Instituto de Salud Carlos III”), San Sebastian-Donostia, Spain; 14 IKERBASQUE, Basque Foundation for Science, Bilbao, Spain; 15 Department of Biochemistry and Genetics, School of Sciences, University of Navarra, Pamplona, Spain; 16 SC Immunologia clinica – Malattie infettive, Fondazione IRCCS Policlinico San Matteo, Viale Golgi 19, 27100 Pavia, Italy; 17 Department of Internal Medicine and Therapeutics, University of Pavia, Pavia, Italy

**Keywords:** Cancer microenvironment, Liver cancer, Sphingolipids, Monocytes and macrophages

## Abstract

Cholangiocarcinoma (CCA) is a rare cancer characterized by a global increasing incidence. Extracellular vesicles (EV) contribute to many of the hallmarks of cancer through transfer of their cargo molecules. The sphingolipid (SPL) profile of intrahepatic CCA (iCCA)-derived EVs was characterized by liquid chromatography-tandem mass spectrometry analysis. The effect of iCCA-derived EVs as mediators of inflammation was assessed on monocytes by flow cytometry. iCCA-derived EVs showed downregulation of all SPL species. Of note, poorly-differentiated iCCA-derived EVs showed a higher ceramide and dihydroceramide content compared with moderately-differentiated iCCA-derived EVs. Of note, higher dihydroceramide content was associated with vascular invasion. Cancer-derived EVs induced the release of pro-inflammatory cytokines in monocytes. Inhibition of synthesis of ceramide with Myriocin, a specific inhibitor of the serine palmitoyl transferase, reduced the pro-inflammatory activity of iCCA-derived EVs, demonstrating a role for ceramide as mediator of inflammation in iCCA. In conclusion, iCCA-derived EVs may promote iCCA progression by exporting the excess of pro-apoptotic and pro-inflammatory ceramides.

## Introduction

Primary liver cancer remains a global health challenge and its incidence is growing worldwide. It is estimated that, by 2025, > 1 million individuals will be affected by liver cancer annually. The two major histological types are hepatocellular carcinoma (HCC), which comprises around 75% of all liver cancer cases, and intrahepatic cholangiocarcinoma (iCCA) accounting for about 12–15%. iCCA, as well as perihilar (pCCA) and distal CCA (dCCA), belong to a heterogeneous group of malignancies occurring at any point of the biliary tree. CCA is a rare cancer, but its incidence (0.3–6 per 100,000 person-years) and mortality (1–6 per 100,000 person-years) have been significantly rising over the last few decades^1^. Risk factors include a variety of conditions which result in inflammation and cholestasis, such as parasitic infections, primary sclerosing cholangitis, biliary duct cysts, cholelithiasis, toxins (including alcohol and tobacco smoking), HCV or HBV infection, cirrhosis, diabetes, obesity and genetic factors. In Southern Asia, CCA has been associated with infection with liver flukes, such as *Opistorchis viverrini* and *Clonorchis sinensis*^1,2^.

Communication between cancer cells and the tumor microenvironment is a crucial process in cancer evolution and progression. Such crosstalk occurs via direct cell–cell contact, as well as secretion and uptake of soluble cytokines and growth factors. The extracellular vesicle (EV) trafficking has emerged as another important mechanism of cell–cell communication. EVs are particles naturally released from the cell that are delimited by a lipid bilayer and cannot replicate. EVs include small EVs and medium/large EVs, with specific ranges defined as <100 nm or  < 200 nm (small), or  > 200 nm (large and/or medium), respectively^3^. They contribute to many of the hallmarks of cancer, including cell proliferation and migration, angiogenesis, evasion of cell death, invasion and metastasis, mainly through transfer of their bioactive content which can include oncoproteins, oncogenes, lipids, as well as soluble factors, protein transcripts and miRNAs^4^. Additionally, tumor-derived EV can modulate the host immune response through various pathways, including performing pro-inflammatory effects^5^. EVs from colon cancer and multiple myeloma cells induced pro-inflammatory cytokine expression and programmed death ligand 1 upregulation in macrophages, creating an inflammatory microenvironment able to support and to enhance tumor progression^6^. Gärtner *et al*. demonstrated that EVs derived from primary squamous head and neck cancer cell lines activated monocytes and induced the secretion of pro‐inflammatory cytokines such as tumor necrosis factor alpha (TNFα) and interleukin (IL)1β^7^. EVs from oral squamous carcinoma cells caused activation of the inflammatory pathway in monocytes via miR-21, contributing to establish a pro-inflammatory and pro-tumorigenic milieu^8^. Moreover, monocytes activated by cancer-derived-EVs increased HLA-DR expression, reactive oxygen intermediate production, accumulation of mRNA and secretion of TNF, IL-10, IL-12p40^9^. In contrast, transfer of cancer-derived microparticles to monocytes was found to change their cytokine profile towards a reduced release of the pro-inflammatory cytokines GM-CSF and TNF-α and an increased release of the anti-inflammatory cytokine IL-10^10^. Interestingly, in triple negative breast cancer, cancer-derived EVs promoted monocyte differentiation into a subset of proinflammatory monocyte-derived macrophages^11^, and EVs specifically promoted proinflammatory macrophages bearing an interferon response signature^11^. Importantly, EVs are also key mediators of hepatic inflammation which characterizeses virtually all liver diseases, including non-alcoholic fatty liver disease (NAFLD), acute liver injury, alcoholic hepatitis, chronic viral hepatitis, HCC and cholangiopathies^12^. CCA-derived EVs found in patients’ sera, bile and urine feature specific RNA and protein profiles mirroring the tumor, envisioning a possible non-invasive approach for the diagnosis of CCA^13–15^. Moreover, CCA cell-derived EVs carry a specific cargo that has been associated with dysregulated proliferation, migration and invasion of tumor cells^12,16,17^. Though protein and RNA content have been investigated in liver cancers, the lipid profile of CCA-derived EVs is still largely unknown.

Sphingolipids (SPL) are a broad class of membrane components and signaling mediators involved in a variety of pathological processes, namely those that are related to inflammatory responses or inflammation-associated diseases. SPLs also regulate cell growth, proliferation, migration, invasion, and metastasis by controlling signaling functions within the cancer cell signal transduction network^18,19^. In particular, ceramide (Cer) and its metabolite ceramide 1-phosphate play key roles in a number of inflammation-associated diseases such as cystic fibrosis^20,21^, lung cancer^22^ and NAFLD^23,24^. SPL metabolism is widely dysregulated in chronic liver disease and liver cancer^19,25^, while Cer and sphingomyelin (SM) content undergoes important remodeling in various CCA cell lines^25^.

In this study, we investigated the SPL profile by liquid chromatography-tandem mass spectrometry (LC–MS/MS) analysis of EVs isolated from supernatants of primary iCCA cells and from normal human cholangiocytes (NHC). We detected an altered SPL content in EVs derived from primary iCCA cells and an enrichment of Cer and dihydroceramides (DHCer) in poorly-differentiated iCCA-derived EVs. Moreover, we showed that iCCA-derived EVs are mediators of inflammation inducing expression of IL-1α, IL-8 and macrophage inflammatory protein-1 alpha (MIP-1α) in monocytes via Cer.

## Results

### SPL concentration was reduced in primary iCCA-derived EVs

**Figure 1:**
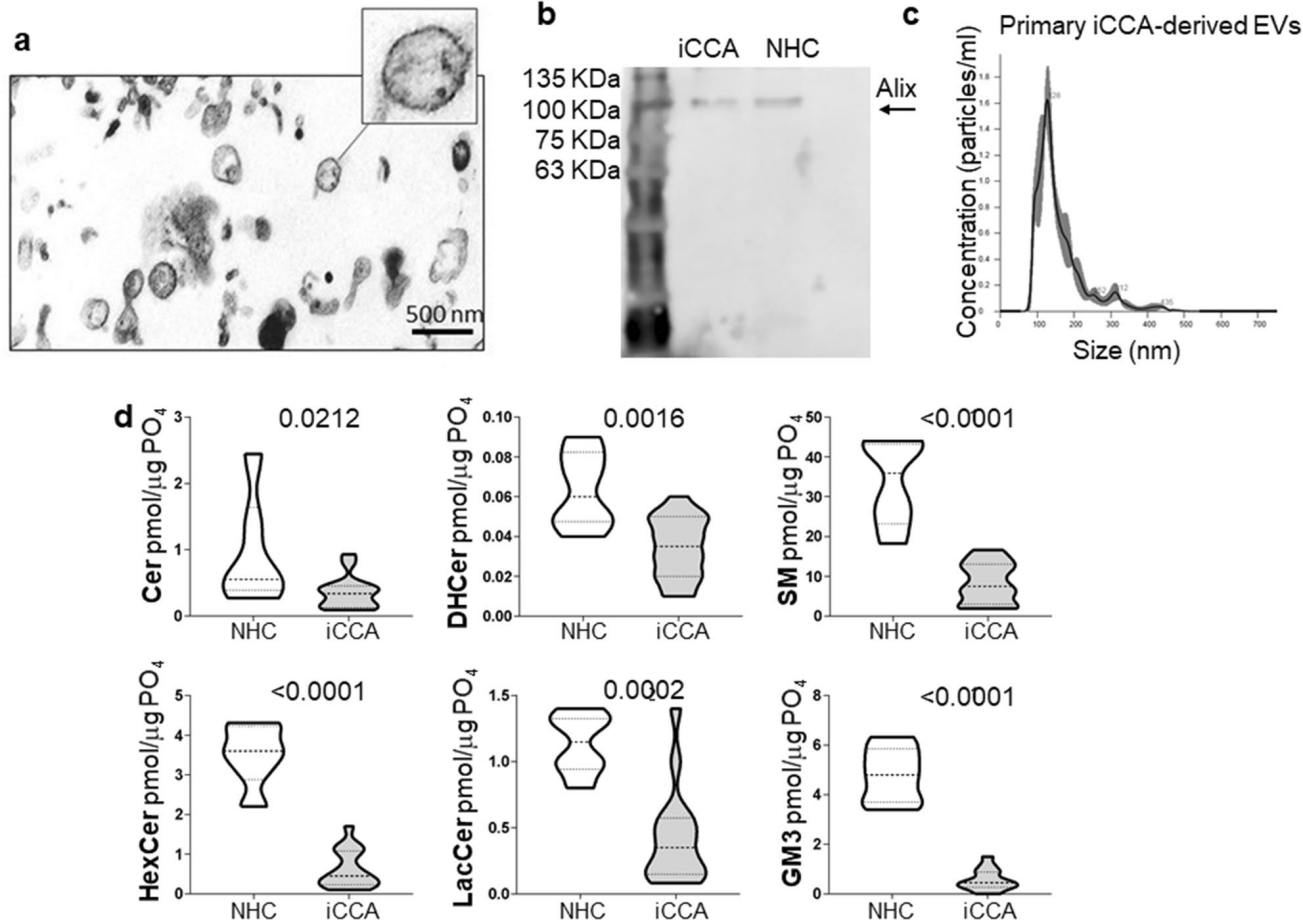
SPL concentration was downregulated in primary iCCA cell-derived EVs. (**a**) Representative transmission electron micrograph of primary iCCA-derived EVs. Scale bar = 500 nm. (**b**) Representative image showing a Western blot of Alix (lane 1 = standard ladders; lane 2 = primary iCCA-derived EVs; lane 3 = NHC-derived EVs). The image has been cropped and the original, uncropped blots at multiple exposures (3, 10 and 15 s) are presented in Supplementary Fig. 6. (**c**) Size analysis of primary iCCA-derived EVs was determined by Nanoparticle Tracking Analysis. (**d**) Analysis of the different SPL classes in supernatant-derived EVs from iCCA and NHC cells by liquid chromatography-tandem mass spectrometry (iCCA = 16; NHC = 6). Median values and interquartile ranges are shown in the violin plots. The unpaired t test was used to compare data, except for Cer for which the Mann–Whitney U test was used.

Primary cell cultures were established from patients affected by iCCA who underwent tumor resection and whose characteristics are listed in Table 1. Cell cultures were characterized for their expression of the biliary marker cytokeratin 19 (CK19) and all expressed CK19 (Supplementary Fig. 1). Supernatant-derived EVs from primary iCCA and from NHC cell cultures were isolated by ultracentrifugation and characterized for their morphology, size, marker and lipid profile. The analysis performed by transmission electron microscopy (TEM) revealed a round-shape morphology of iCCA-derived EVs (Fig. 1A), while the immunoblotting showed a high concentration of the exosome protein marker Alix (Fig. 1B) and CD81 (Supplementary Fig. 2). The determination of iCCA- and NHC-derived EVs size was assessed by nanoparticle tracking analysis (NTA, Fig. 1C) and dynamic light scattering (DLS, supplementary Figure 3) and displayed an average diameter of 160.0 ± 4.1 nm and of 145.1 ± 1.1 nm, respectively, with a polydispersity index (PDI) of 0.2, suggesting that they were a heterogeneous population of small EVs. To establish the SPL profile, a LC–MS/MS analysis was performed. As shown in Fig. 1D, downregulation of all SPL species was observed in iCCA-derived EVs. Indeed, concentrations of Cer, DHCer, SM, hexosylceramides (HexCer), lactosylceramides (LacCer) and gangliosides (GM3) were lower in iCCA- than in NHC-derived EVs.

**Table 1:** Clinical characteristics of patients.

	iCCA	%
Number of patients	16	
Male/Female	13/3	81.25/18.75
Age (years) median, range	69 (56–81)	–
ALT (mU/ml) median, range	20 (11–86)	–
AST (mU/ml), median, range	25 (17–70)	–
PLT (10^3^/ul), median, range	248 (126–351)	–
Glucose (mg/dl), median, range	104 (91–163)	–
TG (mg/dl), median, range	97 (43–204)	–
CHOL (mg/dl), median, range	190 (84–324)	–
HDL (mg/dl), median, range	50 (22–92)	–
LDL (mg/dl), median, range	112 (59–253)	–
Alb (g/dl), median, range	4.3 (2.6–48.3)	–
Tot Bil (mg/dl), median, range	0.8 (0.4–1.9)	–
CA 19.9 (IU/ml), median, range	37 (4.8–6470)	–
BMI: 18.5–24.99	5	31.25
25.00–29.99	4	25
0.00–34.99	5	31.25
NA	2	12.5
DM2 (Yes/No)	7/9	43.7/56.3
Dyslipidemia (Yes/No)	8/8	50/50
Cirrhosis (Yes/No)	1/15	6.2/93.8
Vascular Invasion (Yes/No/NA)	5/10/1	31.25/62.5/6.25
Tumor size (cm): < 2	2	12.5
2–5	7	43.75
> 5	7	43.75
Tumor Grading: G2	8	50
G3	8	50
Tumor Stage: T1	4	25
T2	10	62.5
T3	2	12.5
Etiology: HBV	1	6.25
NASH	5	31.25
Unknown	10	62.5

ALT, alanine aminotransferase; AST, aspartate aminotransferase; PLT, platelet; TG, triglyceride; CHOL, cholesterol; HDL, high-density lipoprotein; LDL, low-density Lipoprotein; Alb, albumin; Tot Bil, total bilirubin; CA19.9, carbohydrate antigen 19–9; BMI, body mass index; NA, not available; DM2, type 2 diabetes mellitus; HBV, hepatitis B virus; NASH, nonalcoholic steatohepatitis.

### Cer and DHCer were enriched in EVs derived from poorly-differentiated iCCA

**Figure 2:**
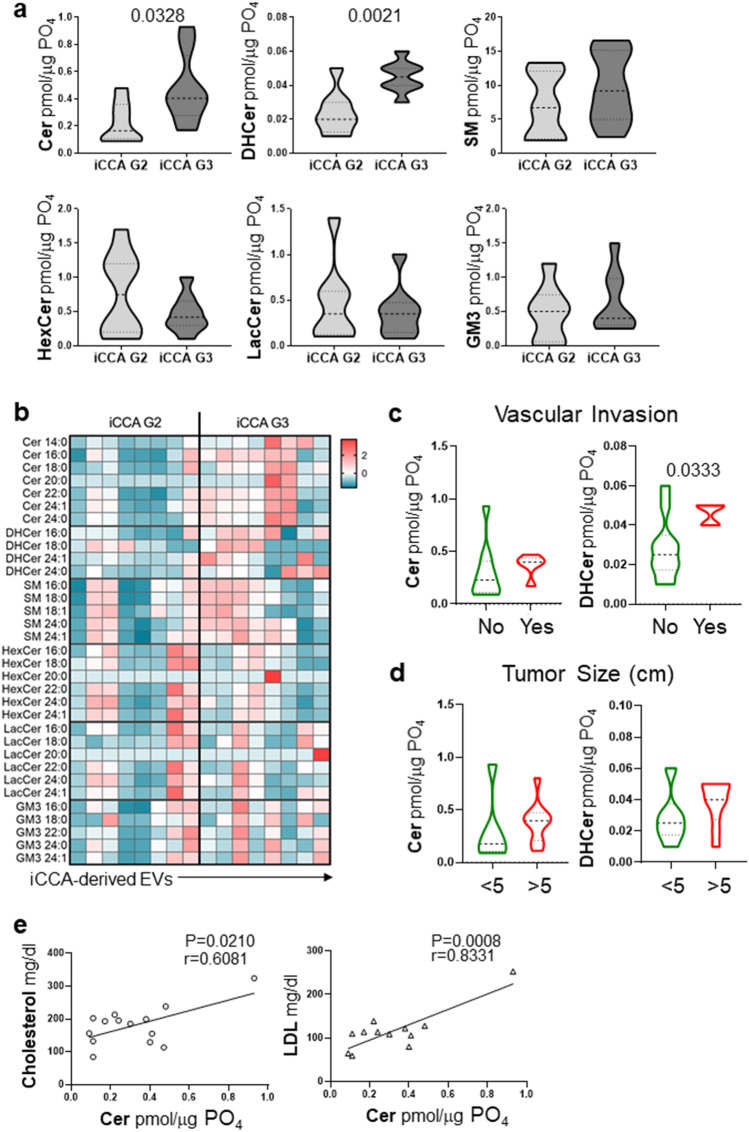
Cer and DHCer expression in primary iCCA-derived EVs changed according to tumor grade. (**a**) Analysis of the SPLs in primary iCCA cell-derived EVs isolated from patients with iCCA stratified according to tumor grade. G2, moderately-differentiated tumors (n = 8); G3, poorly-differentiated tumors (n = 8). Median values and interquartile ranges are shown in the violin plots. The unpaired t test was used to compare data. (**b**) Heatmap of the main SPL species in G2- compared with G3-derived EVs. Each column represents EVs derived from primary iCCA cells obtained from each patient. The entity of the fold change has been visualized by color gradient from the smallest (cerulean) to the highest (red) values, passing through the baseline (white). Data has been log transformed and auto-scaled obtaining z-score prior to visualization. (**c** and **d**) Analysis of Cer and DHCer in EVs from patients stratified according to the presence of vascular invasion or tumor size, respectively. Vascular invasion was histologically defined by tumor cells infiltrating vessel walls with associated thrombi, or intravascular cancer cells mixed with thrombi. Median values and interquartile ranges are shown in the violin plots. The unpaired t test was used to compare data. (**e**) Significant correlations between cholesterol or LDL plasma levels and EV-Cer content.

When iCCA samples were stratified according to tumor grade, we showed that all SPL species were downregulated in EVs derived from both moderately- (G2) and poorly-differentiated (G3) tumors compared with NHC, except for Cer that was decreased in G2 samples only (Supplementary Fig. 4). Interestingly, Cer and DHCer species were higher in G3- compared to G2-derived EVs (Fig. 2A). Indeed, Cer and DHCer concentrations in G3-derived EVs were almost twice that of G2 EVs, increasing from mean values of 0.225 ± 0.05 to 0.476 ± 0.09 and 0.023 ± 0.004 to 0.045 ± 0.008 pmol/μg PO_4_, respectively. Conversely, we noted in G3-derived EVs a trend towards a reduction in HexCer and LacCer concentrations by approximately 40% and 20%, respectively. SM and GM3, were both slightly increased in G3-derived EVs. Of note, a higher concentration of DHCer in EVs was associated with tumor vascular invasion with a trend for Cer towards a higher, though not significant, content (Fig. 2C). Also, a trend towards a higher Cer and DHCer content was associated with larger tumor size (Fig. 2D). Moreover, higher levels of Cer correlated with plasma cholesterol and low-density lipoprotein (LDL) levels (Fig. 2E). All SPL species were represented across samples (fatty acid chain between C16–C24) as shown in the heatmap (Fig. 2B). G3-derived EVs showed an increase in the concentrations of Cer (fold-change, FC 2.54), DHCer (FC 1.74), SM (FC 1.26) and simple gangliosides (GM3, FC 1.38). This trend was restricted to long chain and very long chain SPLs (C > 20) such as Cer 20:0 (FC 5.66), Cer 24:0 (FC 2.49), DHCer 24:0 (FC 2.01), SM 24:0 (FC 1.48) and GM3 24:1 (FC 1.47). In contrast, G3-derived EVs revealed a reduced concentration of simple neutral glycosphingolipids such as HexCer (FC 0.64) and LacCer (FC 0.88). This was also shown on very long chain glycosphingolipids such as HexCer 24:1 (FC 0.61) and LacCer 22:0 (FC 0.75).

### ICCA-derived EVs induced pro-inflammatory cytokine secretion by monocytes via ceramide

**Figure 3:**
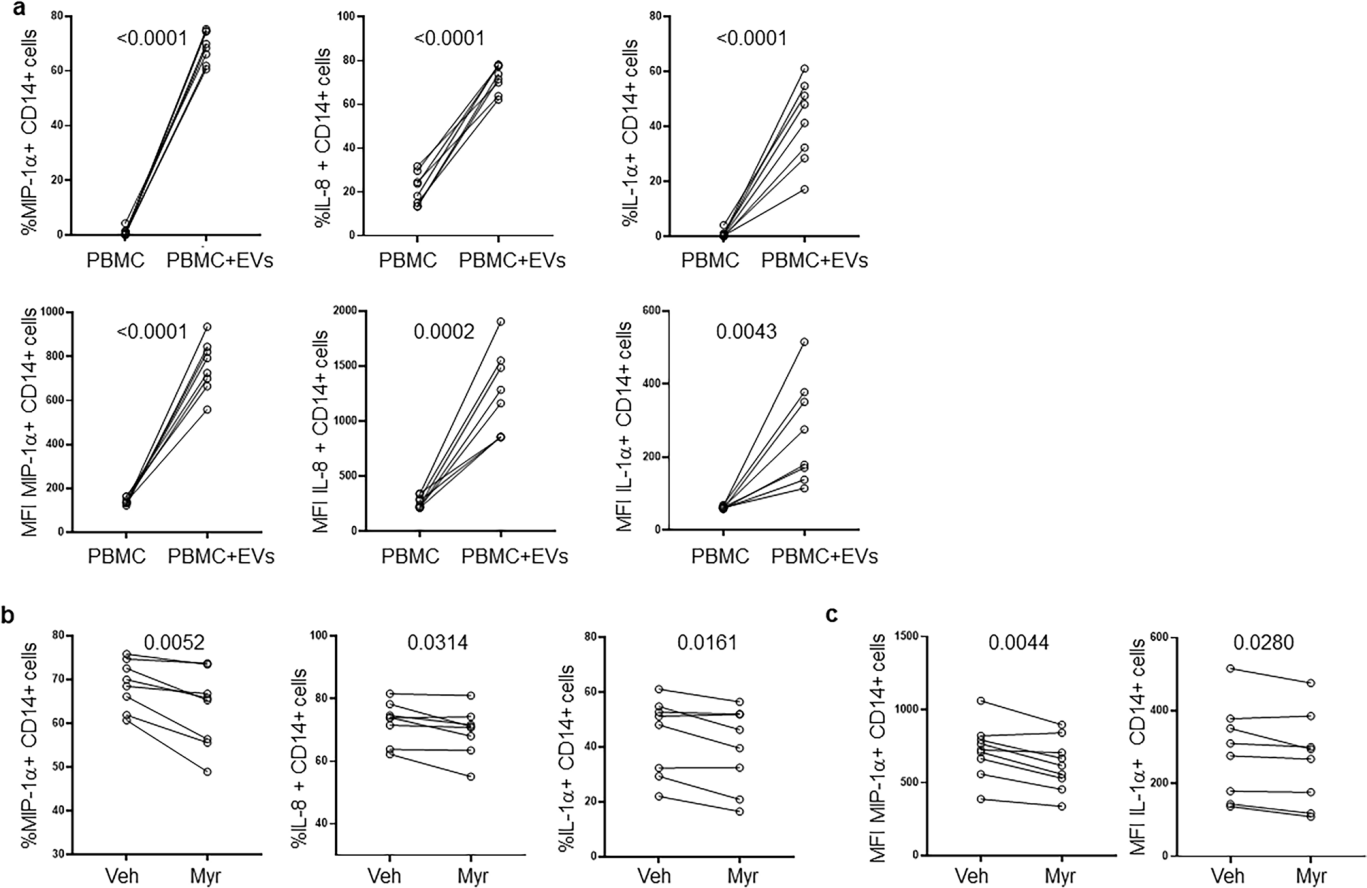
iCCA-derived EVs induce pro-inflammatory cytokine expression in monocytes. (**a**) PBMC from healthy controls (n = 8) were treated with HuCCT-1-derived EVs for 16 h before flow cytometric analysis. Percentage (upper panels) and expression (lower panels, mean fluorescence intensity, MFI) of MIP-1α-, IL-8- and IL-1α-CD14 + cells are reported. The paired t test was used to compare data. (**b**) Percentage of MIP-1α-, IL-8- and IL-1α-CD14 + cells after treatment for 16 h of PBMC with EVs exhibiting a different Cer content. EVs were isolated from HuCCT-1 cells after treatment with myriocin (Myr) at 10 μM and DMSO (Veh) for 16 h before collecting supernatants. The paired t test was used to compare data. (**c**) Expression (MFI) of MIP-1α and IL-1α in CD14 + cells after treatment of PBMC with EVs. The paired t test was used to compare data.

Since Cer is widely involved in inflammatory processes, we tested the ability of iCCA-derived EVs to induce a pro-inflammatory response in monocytes. To this end, supernatant-derived EVs from the iCCA cell line HuCCT-1 were used to treat peripheral blood mononuclear cells (PBMC) isolated from healthy controls (HC). The gating strategy to analyze monocytes is described in Supplementary Fig. 5. As shown in Fig. 3A, EVs induced a strong increase in the proportion of MIP-1α-, IL-8- and IL-1α-CD14+ cells compared with untreated PBMC. Expression of the same cytokines on CD14+ cells, as shown by mean fluorescence intensity (MFI), was also increased (Fig. 3A, lower panels). To test whether the pro-inflammatory effect of EVs on monocytes was mediated by their Cer content, we treated HuCCT-1 cells with Myriocin (Myr), a specific inhibitor of the serine palmitoyl transferase, the first enzyme involved in the *de novo* synthesis of Cer, or with the vehicle (DMSO) before isolation of EVs from supernatants. LC–MS/MS analysis of EVs demonstrated that Myr treatment reduced the content of Cer to 6.4 pmol/µg PO_4_ from the 16.4 pmol/µg PO_4_ measured in EVs derived from DMSO-treated HuCCT-1 cells. EVs showing different Cer content were used to treat PBMC. Cer-deprived EVs isolated from Myr-treated HuCCT-1 cells decreased the proportion of MIP-1α-, IL-8- and IL-1α-CD14+ cells (Fig. 3B) and expression (MFI) of MIP-1α and IL-1α in monocytes (Fig. 3C), demonstrating the involvement of Cer in inducing a pro-inflammatory status of monocytes in this setting.

## Discussion

Tumor-derived EV and EV-related cargo have clearly revealed their essential functional role in modulating hallmarks of cancer such as tumor angiogenesis, tumor stroma formation, tumor development and tumor proliferation and invasion^26^. In CCA, the few studies that evaluated EV cargo focused on their protein and nucleic acid content, highlighting the promising role of EVs as a non-invasive tool for diagnosis^14,15^, as well as its functional role in tumor proliferation and invasion. CCA-derived EVs induced the differentiation of bone marrow-derived mesenchymal stem cells into fibroblasts releasing cytokines and chemokines that stimulated CCA growth^16^. Moreover, CCA cell line KKU-M213-derived EVs promoted migration and invasion of SV40-immortalized human cholangiocytes (H69 cells), consistent with the proteomic analysis showing the enrichment of integrin and glycoproteins in EVs derived from CCA cells^17^. However, the lipid cargo of CCA-derived EVs, and in particular the SPL content, has only been scarcely analyzed.

In this study, we profiled SPLs from supernatant-derived EVs isolated from primary iCCA cells. SPLs play a relevant role in tumorigenesis since they are essential building blocks of plasma membranes and participate in the composition of lipid rafts that drive pro-tumor signaling^27^. The overall decrease of SPL levels in iCCA-EVs compared with normal cholangiocyte-derived EVs highlights the relevant pro-tumorigenic role of these lipids that may be retained within tumor cells for their survival^28,29^. SPLs comprise a wide number of molecules, whose central mediator is Cer, which may be converted into more complex SPLs (SM, HexCer, LacCer, GM3) and in turn reversibly released from each of them by the action of catabolic enzymes in response to cellular needs^18^. Of note, the core finding of our study concerns the expression of Cer in iCCA-derived EVs. In iCCA samples stratified according to tumor grade, we observed that Cer and its precursor DHCer, are predominantly accumulated in G3- compared with G2-released EVs. Moreover, G3-released EVs are enriched in long chain and very long chain SPLs species. Cer is a recognized mediator of anti-proliferative and death signaling pathways and participates in processes such as apoptosis^30,31^. The removal of this bioactive lipid from the more aggressive and proliferative G3 tumor through EVs, could therefore represent an adaptive mechanism to promote resistance to cell death. In addition, Ali *et al.* showed that altering the acyl chain composition of SPLs decreased very long chain SPLs, and inhibited TNF-α receptor-1 internalization and TNF-α pro-apoptotic downstream signaling. In this way, iCCA-G3 exports with EVs the long chain and very long chain SPLs promoting tumor aggressiveness as well as driving away the pro-apoptotic signaling^32^. Accordingly, we observed that the higher expression of Cer and DHCer in EVs, correlated with the presence of vascular invasion and a larger tumor mass.

Furthermore, a positive correlation was found between Cer content in EVs and cholesterol and LDL plasma levels. Several studies have demonstrated that a high-fat, high cholesterol diet can trigger HCC in mice, via neoplastic transformation of hepatocytes caused by broad transcriptional deregulation of genes involved in metabolic pathways. Of note, it has been shown that statins, which block hepatic cholesterol synthesis, protect against HCC and CCA development, and decreased mortality^19,33^. On the other hand, serum cholesterol levels in patients with iCCA were found to be unchanged^34^. The lack of adequate information on CCA lipidomic is reflected by the existence of only a few studies currently limited to biomarker discovery. Thus, a comprehensive investigation of the CCA lipidome landscapes is still lacking^19^. The positive correlation between EV-associated Cer and cholesterol/LDL plasma levels requires additional in-depth studies to elucidate whether an excess LDL/cholesterol could influence intratumoral cell Cer pathway and release.

Cer holds a relevant role in a number of inflammatory disorders^20–24,35,36^, however, there is only a scarcity of information on its role in tumor-derived EVs. Interestingly, in a different disease setting such as cystic fibrosis, EVs transport the excess of Cer out of the cell thus preventing resolution of pulmonary inflammation^20,21^. Here we showed that Cer excess was exported by G3-derived EVs, suggesting a possible role in iCCA growth by maintaining an inflamed microenvironment. Indeed, following treatment with Cer-rich EVs, monocytes were activated and exhibited a pro-inflammatory phenotype that was restored by Myr treatment. Indeed, by inhibiting the rate-limiting step of the Cer biosynthetic pathway, Myr reduces Cer accumulation and in turn Cer-driven inflammation, as described in several pathological conditions^21,37–39^.

Concerning the energy metabolism, Cer may be conjugated with glucose residues to generate glycated ceramides and then released through catabolic reactions. Notably, we observed a trend towards a reduction in HexCer and LacCer expression in G3 compared with G2 iCCA-derived EVs. Like other tumors, CCA is highly dependent on lipid and glucose metabolism^25,40^. Thus, similarly to the highly proliferative CCA cell lines, that are dependent upon increased lipid uptake and metabolization via fatty acid oxidation^25^, poorly-differentiated tumors may require more glucose than the moderately-differentiated ones to sustain their metabolic demand. Therefore, the degradation of glycated ceramides could represent an additional source of glucose which could account for the accumulation of Cer, eventually removed by EVs.

Serum metabolomic profiling of patients with iCCA and controls have identified a total of 52 altered metabolites, including SM, in patients with iCCA^34^. Interestingly, decreased SM was one of the nine altered metabolites showing higher diagnostic accuracy for iCCA than CA19-9, commonly used in the diagnosis of iCCA^34^. In agreement with this study, we found that iCCA cells release EVs containing low levels of SM.

In conclusion, EVs secreted by poorly-differentiated tumor cells may facilitate tumor progression by exporting the excess of pro-apoptotic and pro-inflammatory Cer. This study provides novel findings on altered SPL expression in iCCA-derived EVs, setting the stage for further extensive analysis contributing to unveil the role of lipids in the tumorigenic process.

## Materials and methods

### Study subjects

Surgically resected iCCA specimens were obtained from patients admitted to Fondazione IRCCS Policlinico San Matteo, Pavia, IRCCS Humanitas Research Hospital, and ASST Santi Paolo e Carlo Hospital, Milan, Italy. The main patient characteristics are listed in Table 1.

PBMC were isolated as previously described^41^ from HC (3 females and 5 males). A written informed consent was obtained from each individual. The study was conducted in accordance with the Declaration of Helsinki, and approved by the Ethics Committee of Fondazione IRCCS Policlinico San Matteo, Pavia (protocol numbers: P-20140031379, P-20190104922).

### Cell cultures

NHC cells were isolated from normal liver tissue specimens and maintained as previously described^25^.

Primary tumor cell cultures were established from patients affected by iCCA as previously described^41^. Briefly, tumor samples were treated by enzymatic and mechanical dissociation with the human Tumor Dissociation Kit and gentleMACS Dissociator (Miltenyi Biotec, Bergisch Gladbach, Germany), according to the manufacturer's instructions. The cell suspension was filtered and centrifuged to obtain a cell pellet that was plated in tissue culture flasks (Corning, NY, USA) with Dulbecco’s Modified Eagle Medium (Thermo Fisher Scientific, Waltham, MA, USA) supplemented with 10% fetal bovine serum (FBS, HyClone, GE Healthcare, South Logan, Utah, USA), 1% antibiotic antimycotic solution (Merck, Darmstadt, Germany) and 1% non-essential amino acids (Thermo Fisher Scientific).

CK19 was evaluated in primary iCCA cell cultures by flow cytometry. Briefly, iCCA cells were fixed with BD Cytofix/Cytoperm (BD Biosciences, San Diego, CA, USA) and permeabilized with the BD Perm/Wash buffer (BD Biosciences) in the presence of FITC mouse anti-human monoclonal antibody (mAb) (clone SB39 g, Abcam, Cambridge, UK) for 30 min at 4 °C, according to the manufacturer’s instructions. Flow cytometry analysis was performed using a 12-color FACSCelesta (BD Biosciences) instrument. Kaluza™ software (Beckman Coulter, Brea, CA, USA) was used for data analysis. HuCCT-1 cells (kindly provided by professor M. Cadamuro, Dept. of Molecular Medicine, University of Padua, Italy) were maintained as previously described^41^.

### EV isolation and characterization

Supernatants from low-passage primary iCCA and NHC cells were collected after 72 h of culture in medium without serum, bovine pituitary extract and lipid and cryopreserved at −80 °C until EV isolation. Supernatants from HuCCT-1 cells were collected after 16 h of culture in medium without serum with 0.8% bovine serum albumin (Merck). When indicated, HuCCT-1 cells were treated with 10 μM Myr (Merck) and DMSO (Merck) for 16 h before collecting supernatants to isolate EVs. After serial centrifugations (1000, 2000 and 3000 g for 10 min at 4 °C), EVs were obtained by ultracentrifugation (100,000 g for 75 min at 4 °C). EVs were then resuspended in PBS with protease inhibitors and stored at −20 °C until LC–MS/MS evaluation. Freshly isolated EVs from HuCCT-1 cells were resuspended in PBS and used to treat PBMC. EV characterization was performed by NTA, DLS, TEM and Western Blotting, as previously described^20^. Briefly, NTA was carried out with the Malvern NanoSight NS300 system (Malvern Panalytical ltd., Malvern, UK), used to visualize EVs by laser light scattering. For each sample, three 60-sec records were registered. NTA output was then analyzed with integrated NTA software (Malvern Panalytical ltd.), providing high-resolution particle size distribution profiles and EV concentration measurements. DLS measurements were performed on a home-made apparatus, equipped with four detectors at 90° suitable for diluted samples. The intensity correlation functions were analyzed with NNLS and cumulant analysis, to obtain both the mean size of EVs and their size distribution. For TEM, samples were fixed in 2.5% glutaraldehyde in 0.13 M phosphate buffer pH 7.2–7.4 for 2 h, post-fixed in 1% osmium tetroxide, dehydrated and embedded in epoxy resin. A Jeol JEM 1010 transmission electron microscope (Jeol, Tokyo, Japan) was used. Anti-Alix (1:1000, 3A9, ab117600, Abcam, Cambridge, MA, USA) and anti-CD81 antibodies (1:1000, D502Q, #10037, Cell Signaling, Danvers, MA, USA) were used.

### Characterization of sphingolipids by LC–MS/MS

SPL extraction and analysis were performed as previously described^20^. Briefly, SPLs were extracted with a cold methanol/chloroform mixture (850 µL, 2:1, v/v) coupled with alkaline methanolysis (75 µL KOH 1 M, 2 h at 38 °C). The lipid content was normalized to the inorganic phosphorus amount that was determined by the phosphomolybdate and malachite green assay^42^. Briefly, for the inorganic phosphorus determination an aliquot (10 µL) of the lipid extract was mineralized in concentrated perchloric acid (50 µL, 100 °C, >1h) and added with a solution made of 4.2% ammonium molybdate in 5M HCl/0.2% malachite green/H_2_O (700 µL, 1:1:5, v/v/v). The complex of phosphomolybdate and malachite green was detected and quantified by spectrophotometry at 630 nm^42^. The LC-MS/MS consisted of an LC Dionex 3000 UltiMate (Thermo Fisher Scientific) coupled to a tandem mass spectrometer AB Sciex 3200 QTRAP (AB Sciex, Concord, ON, Canada) equipped with electrospray ionization TurboIonSpray™ source operating in positive mode (ESI+).

### PBMC treatment

PBMC were cultured in RPMI supplemented with 10% FBS, 1% antibiotic antimycotic solution and 1% glutamine (Merck) for 16h in the presence of BD GolgiPlug™ Protein Transport Inhibitor (BD Biosciences). When indicated, PBMC were treated with HuCCT-1-derived EVs at a PBMC:EVs ratio of 1:50 for 16 h. After incubation, 5 × 10^5^ PBMC were harvested and stained with anti-human CD14 BB700, CD3 BV421, HLA-DR BV605 and CD16 APC-H7 mAbs (all from BD Biosciences) for 30 min at 4 °C. Cells were subsequently fixed with BD Cytofix/Cytoperm (BD Biosciences) and permeabilized with the BD Perm/Wash buffer (BD Biosciences) in the presence of anti-human MIP-1α FITC, IL-1α PE and IL-8 BV510 mAbs according to the manufacturer’s instructions. FACSCelesta (BD Biosciences) instrument and Kaluza™ software (Beckman Coulter) were used for data acquisition and analysis.

### Statistical analysis

This was performed using the GraphPad Prism 8.4.3 software (GraphPad, La Jolla,CA, USA). Data distribution among groups was checked for normality prior to analysis. We used parametric and non-parametric tests as detailed in the legend. A *p* value ≤ 0.05 was deemed statistically significant. Pearson correlation test was used to assess correlations between EV content of SPLs and clinical features of the patients.

The datasets used and/or analyzed during the current study are available from the corresponding author upon reasonable request.

## Supplementary Information

Supplementary Figures.

## Data Availability

The datasets used and/or analysed during the current study are available from the corresponding author on reasonable reques.
