# Supplementary material for: Ceramide present in cholangiocarcinoma-derived extracellular vesicle induces a pro-inflammatory state in monocytes
Source: Sci Rep. 2023 May 12;13:7766. doi: 10.1038/s41598-023-34676-w (PMC10182100; doi:10.1038/s41598-023-34676-w)
Supplement: Supplementary file 1 — Supplementary Figures. [file 41598_2023_34676_MOESM1_ESM.pdf]

a

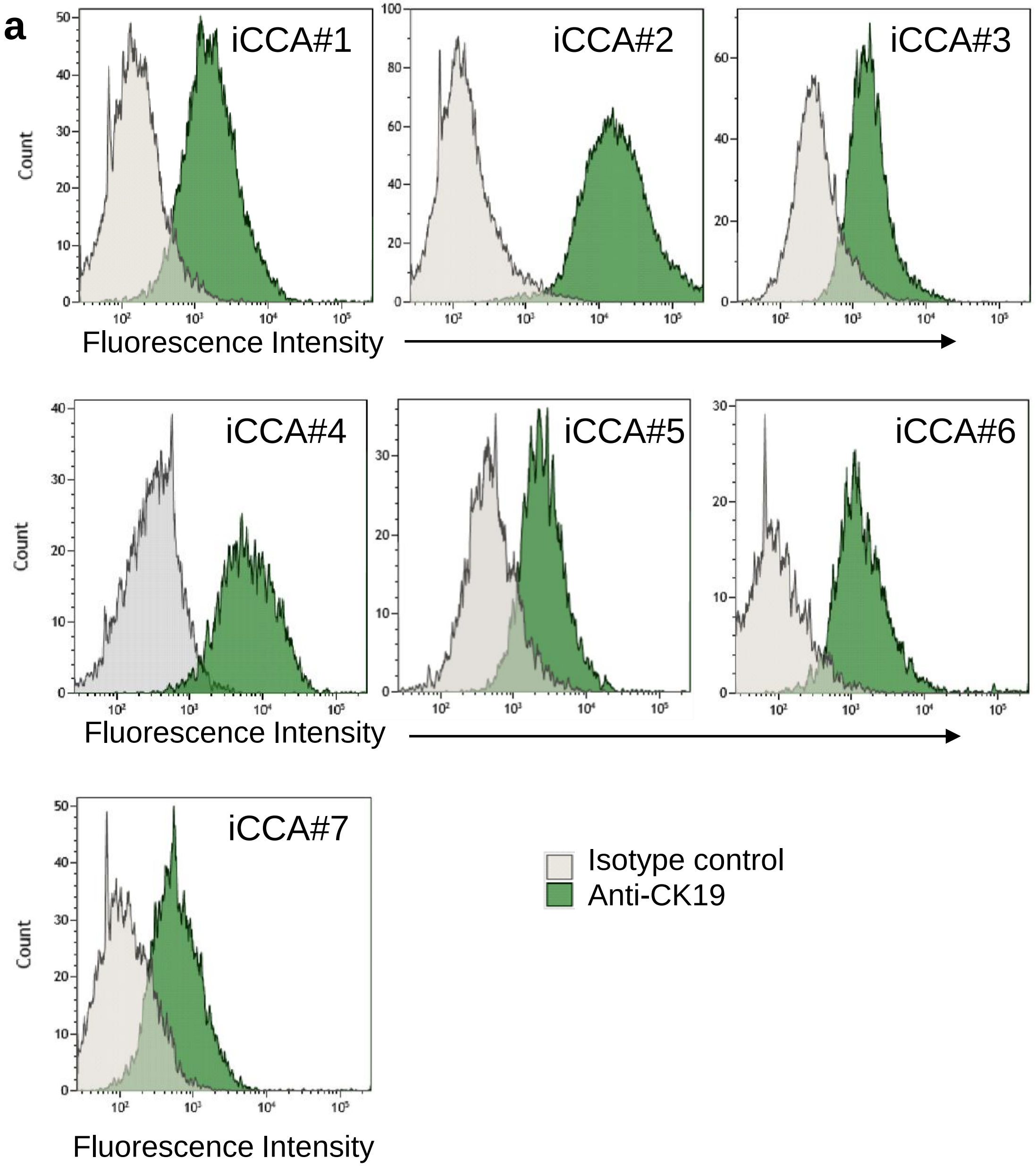

b

Patients	#iCCA1	#iCCA2	#iCCA3	#iCCA4	#iCCA5	#iCCA6	#iCCA7
Male (M), Female (F)	M	M	F	M	M	M	M
Age (years)	71	74	73	76	81	81	65
ALT (mU/ml)	27	38	16	17	27	11	19
AST (mU/ml)	26	27	21	25	32	17	24
PLT (10^3/ul)	234	287	233	180	191	233	304
Glucose (mg/dl)	101	163	94	140	120	108	97
TG (mg/dl)	204	82	70	NA	168	83	124
CHOL (mg/dl)	185	228	132	NA	219	324	129
HDL (mg/dl)	35	70	59	NA	53	54	35
LDL (mg/dl)	109	141	59	NA	132	253	80
Alb (g/dl)	4,2	42,9	4,1	4	4,6	42,6	4
Tot Bil (mg/dl)	0,8	0,7	0,9	1,24	1,9	0,4	0,57
CA 19.9 (IU/ml)	116	51,9	10,2	4,8	32,3	353,3	7,7
BMI	28,34	27,76	24,91	32,9	24,22	31,05	NA
DM2	Y	Y	N	Y	NA	N	Y
Dyslipidemia	N	Y	N	N	Y	Y	N
Cirrhosis	N	N	N	N	N	N	N
Vascular Invasion	N	Y	N	N	Y	N	Y
Tumor size (cm)	3,6	>5	<2	5	2,2	3,7	>5
Tumor Grading	G2	G2	G2	G3	G2	G3	G3
Tumor Stage	T3	T2	T2	T2	T1	T1	T2
Etiology	NA	NASH	NASH	NASH	NASH	NA	HBV

ALT, alanine aminotransferase; AST, aspartate aminotransferase; PLT, platelet; TG, triglyceride; CHOL, cholesterol; HDL, high-density lipoprotein; LDL, low-density Lipoprotein; Alb, albumin; Tot Bil, total bilirubine; CA19.9, carbohydrate antigen 19-9; BMI, body mass index; DM2, type 2 diabetes mellitus; HBV, hepatitis B virus; HCV, Hepatitis C virus; NASH, nonalcoholic steatohepatitis; Y, Yes; N, No; NA, not available.

Supplementary figure 1. (a) Cytokeratin 19 (CK19) expression on representative iCCA patients-derived cell cultures. (b) Clinical characteristics of patients from whom cell cultures tested for CK-19 expression were obtained.

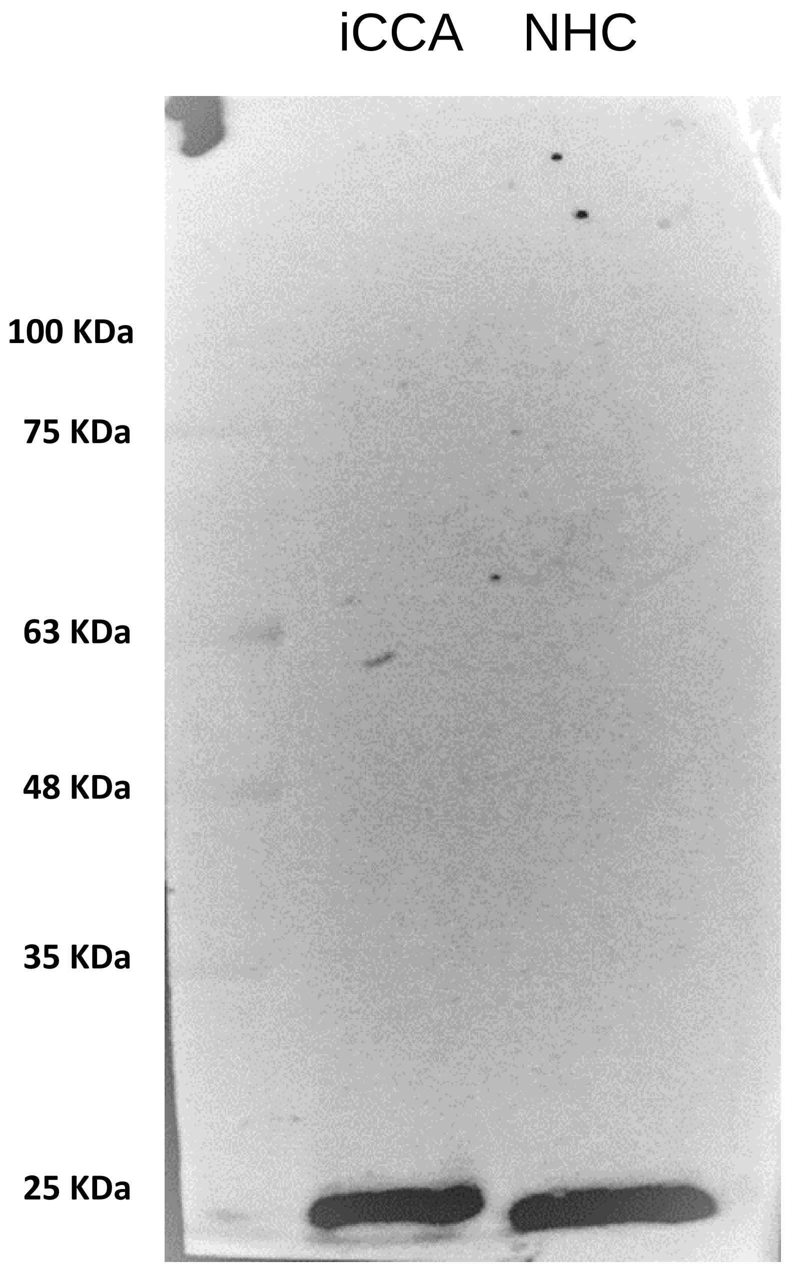

Supplementary Figure 2. Representative image showing a Western blot of CD81 (lane 1= standard MW ladders; lane 2= primary iCCA-derived EVs; lane 3= NHC-derived EVs). CD81 molecular weight is approximately 25 KDa. Anti-CD81, D502Q, #10037, Cell Signaling, 1:1000 was used.

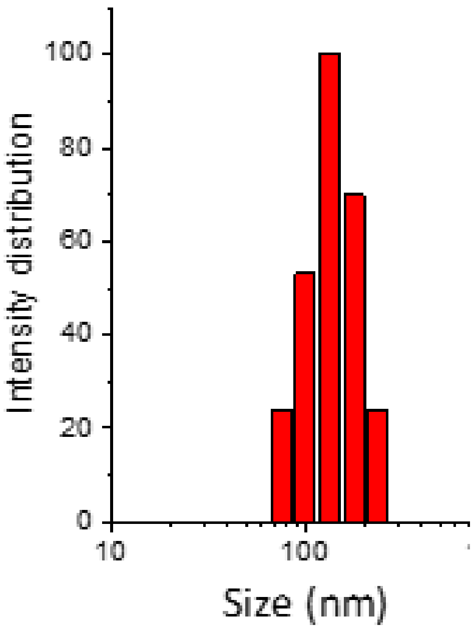

Supplementary Figure 3. A representative image of the size distribution of iCCA-derived EVs as assessed by dynamic light scattering, DLS (Hydrodynamic diameter, intensity weighted).

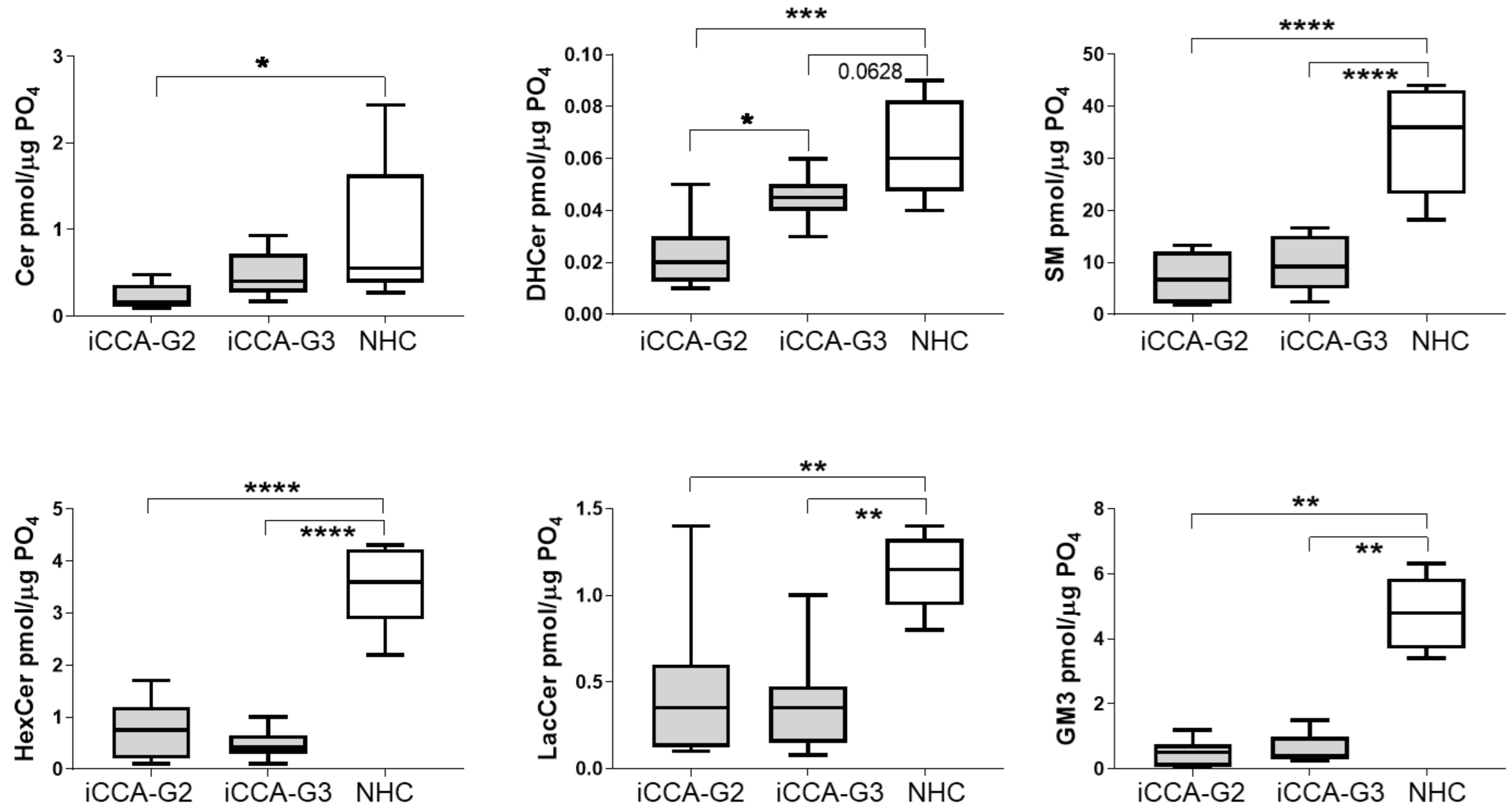

Supplementary Figure 4. EV-SPL expression in iCCA-G2, iCCA-G3 and NHC. iCCA samples were stratified according to tumor grade. G2, moderately-differentiated tumors (n = 8); G3, poorly-differentiated tumors (n = 8). Middle bars represent median values; box plots are 25% and 75% percentiles; whiskers are minimum and maximum values.

*p <0.05, **p <0.01, ***p <0.001, ****p<0.0001. The one-way ANOVA and Tukey's multiple comparisons tests were used to compare data except for GM3 in which Dunn's multiple comparisons test was used.

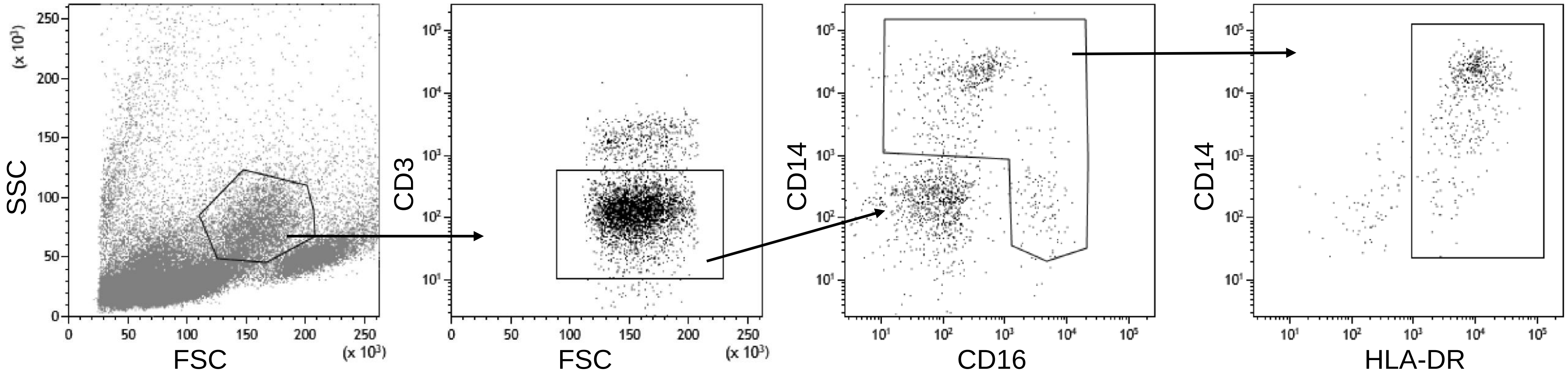

Supplementary Figure 5. Gating strategy adopted for the experiments presented in figure 3.

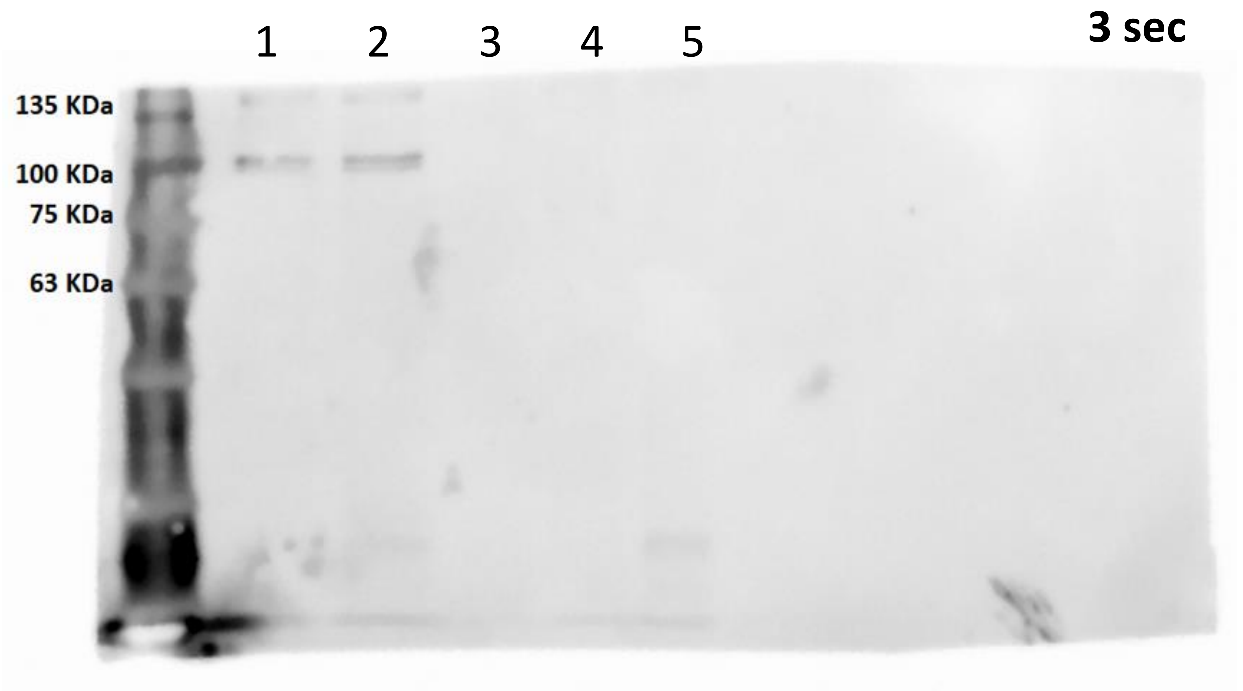

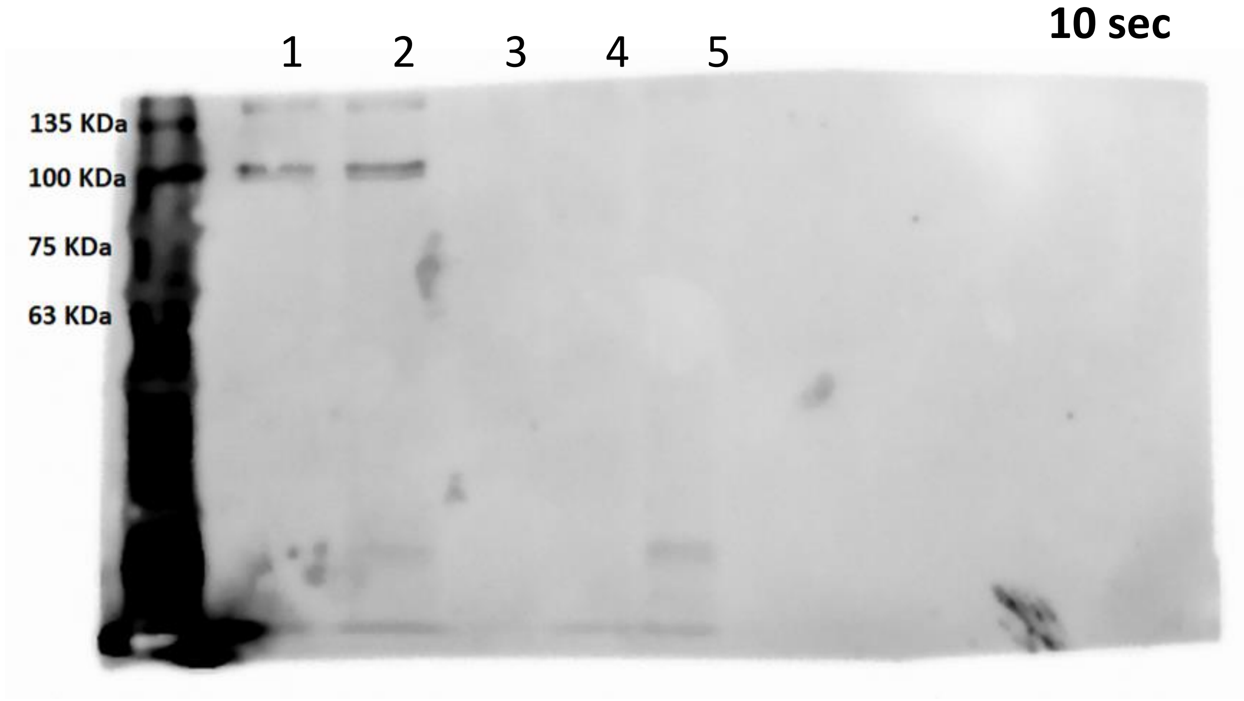

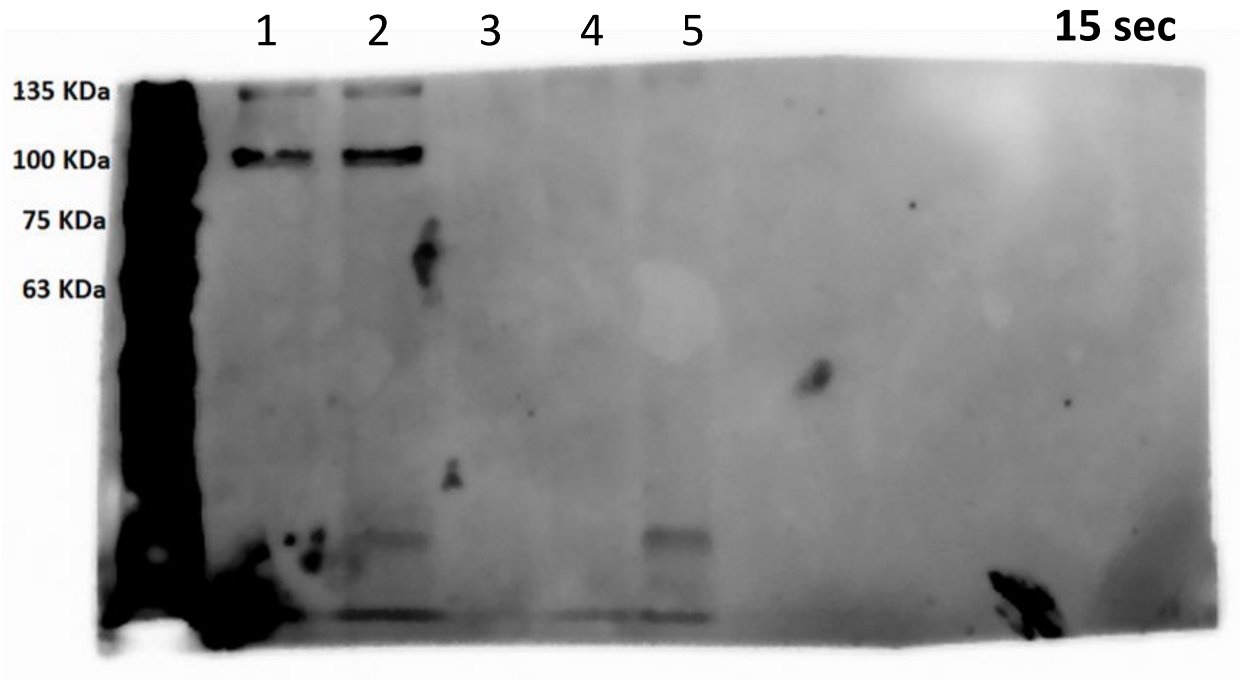

Supplementary Figure 6. Original, uncropped blots shown in figure 1 at multiple exposures (3, 10 and 15 seconds). lane 1= 15 µg protein extract from primary iCCA cell line; lane 2= 15 µ g protein extract from NHC; lanes 3-4 and 5= 5 µg protein extract from primary iCCA cell lines.
